# Lightweight MXene Composite Films with Hollow Egg‐Box Structures: Enhanced Electromagnetic Shielding Performance Beyond Pure MXene

**DOI:** 10.1002/advs.202411932

**Published:** 2025-01-15

**Authors:** Yijie Wang, Chenxiang Zhao, Yunze Tian, Yue Sun, Mengfei Zhang, Kangjing Wang, Bihua Xia, Yang Wang, Ting Li, Xuhui Zhang, Jing Huang, Shibo Wang, Weifu Dong, Jinliang Qiao

**Affiliations:** ^1^ The Key Laboratory of Synthetic and Biological Colloids School of Chemical and Material Engineering Jiangnan University Jiangsu 214122 China; ^2^ SINOPEC Beijing Research Institute of Chemical Industry Beijing 100013 China

**Keywords:** electromagnetic shielding performance, hollow egg‐box structures, MXene composite films

## Abstract

MXene is widely used in the electromagnetic interference (EMI) shielding field. However, the high electromagnetic reflectivity of pure MXene causes potential secondary EMI pollution. This study presents a hollow egg‐box structure used in MXene composite film, by which the reflectivity (R) could decrease from 0.98 to 0.54 and absorbance (A) increased from 0.02 to 0.45, effectively decreasing the high electromagnetic reflectivity of pure MXene. Additionally, compared to pure MXene films, the MXene composite films exhibit improved electromagnetic interference shielding effectiveness (EMI SE) and SSE/t. The prepared films achieve a peak EMI SE of 69.19 dB at 12.4 GHz, which is 1.3 times higher than pure MXene, and a peak SSE/t of 27 888 dB cm^2^ g⁻¹ at 12.4 GHz, 1.4 times that of pure MXene. The hollow egg‐box structure not only enhances the electromagnetic shielding performance beyond pure MXene but also demonstrates outstanding performance compared to most reported MXene films, balancing lightweight material properties with effective shielding. Furthermore, the prepared MXene composite films with the hollow egg‐box structure show improved water resistance. Therefore, MXene composite films with hollow egg‐box structures are promising candidates for advanced EMI devices in future lightweight materials.

## Graphical abstract

This study presents a hollow egg‐box structure used in MXene composite films that could decrease the high electromagnetic wave reflectivity. The prepared films not only show improved electromagnetic interference shielding effectiveness and SSE/t than pure Mxene and most of MXene composite materials, but also show lightweight property and improve water resistance, highlighting their potential for advanced EMI devices in future lightweight materials.

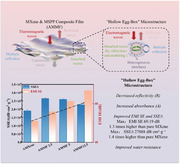

## 1 Introduction

The rapid development of information technology has brought an influx of smart devices, significantly simplifying our lives. However, these devices have also raised concerns about the potential hazards of electromagnetic waves.^[^ ^1^, ^2^, ^3^, ^4^ ^]^ The use of efficient electromagnetic shielding materials can effectively reduce the harm of electromagnetic waves to human health and the environment.^[^ ^5^ ^]^

In recent years, MXene‐based materials have made significant progress in the field of electromagnetic interference (EMI) shielding. As a novel 2D nanomaterial, MXene exhibits great potential in EMI shielding applications due to its high electrical conductivity, high specific surface area, and lightweight properties.^[^ ^6^, ^7^, ^8^ ^]^ However, pure MXene films have a high electromagnetic wave reflectivity and low thickness, leading to insufficient electromagnetic wave absorption, which may result in potential secondary electromagnetic pollution.^[^ ^9^ ^]^ Studies have shown that the electromagnetic wave absorption performance of MXene composites is closely related to their internal structure, in which the complexity and multilevel nature of the structure can significantly optimize the electromagnetic wave propagation path and energy dissipation process.^[^ ^10^ ^]^ As a result, many researchers have attempted to significantly enhance the electromagnetic wave absorption capacity by constructing ultrathin composite films with special microstructures through polymer composite with MXene.^[^ ^11^, ^12^, ^13^ ^]^ For instance, sodium alginate (SA), a polymer with strong interaction with MXene, could prepare MXene composites with high EMI shielding performance and improved electromagnetic wave absorption.^[^ ^3^ ^]^ Additionally, introducing 3D structural materials into MXene has been regarded as an effective approach to enhance EMI shielding performance and electromagnetic wave absorption. Qian et al. embedded cellulose nanofibrils and multiwalled carbon nanotubes into MXene layers to form a multilevel structure, thus improving electromagnetic wave absorption performance.^[^ ^14^ ^]^ Additionally, Aamir and his team prepared MXene composite films by insulating polystyrene microparticles with MXene, effectively improving electromagnetic wave absorption through interface reflection effects.^[^ ^15^ ^]^ As structural design continues to evolve, researchers have shifted their focus to MXene aerogels and foams with honeycomb or porous structures.^[^ ^16^ ^]^ By controlling the size and distribution of pores, the electromagnetic wave propagation path can be optimized, and the scattering effect enhanced, thereby improving the EMI shielding performance.^[^ ^17^ ^]^ Zhang et al. successfully obtained MXene aerogels with uniform porous structures by impregnating MXene solution under vacuum into aerogels, achieving low thermal conductivity, high EMI shielding effectiveness, and excellent electromagnetic wave absorption properties.^[^ ^18^ ^]^

Despite these structural designs significantly improving the electromagnetic wave absorption performance of MXene composites, addressing the issue of potential secondary electromagnetic pollution, achieving sufficient EMI shielding effectiveness often requires high thickness and uses a larger amounts of MXene.^[^ ^19^, ^20^ ^]^ In fields such as aerospace, microelectronics, and wearable electronics, the lightweight nature of materials is particularly crucial. Therefore, a key challenge in current research is how to design microstructures that can simultaneously enhance both the EMI shielding performance and electromagnetic wave absorption capacity of MXene composites, while maintaining their lightweight properties.

Herein, this work reports a kind of MXene composite film with hollow egg‐box structure through vacuum‐assisted self‐assembly and low‐temperature annealing under pressure. The introduction of hollow egg‐box structure significantly alleviates the electromagnetic radiation pollution caused by the high reflectivity of pure MXene films. Notably, even with the addition of fillers, the MXene composite films not only could maintain their lightweight and low‐density characteristics, but also could achieve a maximum electromagnetic interference shielding effectiveness of 69.19 dB, with an SSE/t value of 27 888 dB cm^2^ g^−1^, even showing an improvement compared to pure MXene films. Additionally, the films exhibit good thermal properties and water resistance. Therefore, this work provides a kind of microstructure applied in the development of lightweight and ultrathin MXene‐based film materials, offering broad potential for various applications, such as EMI shielding effects.

## 2 Results and Discussion

**Figure 1:**
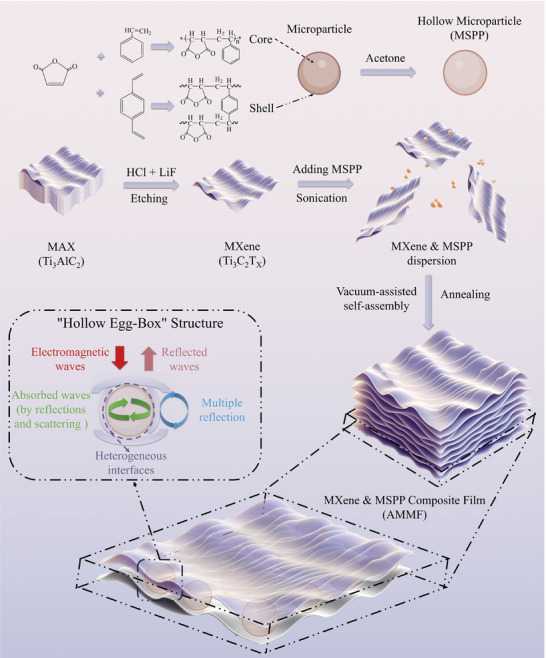
Schematic diagrams of the preparation process of hollow microparticles (MSPP), MXene and MXene&MSPP composite (AMMF) film.

To design microstructures that can simultaneously enhance both the EMI shielding performance and electromagnetic wave absorption capacity of MXene composites film, while maintaining their lightweight properties, this study report a kind of hollow egg‐box structure. By embedding hollow microparticles (MSSPs) between the MXene layers and applying a low‐temperature annealing treatment under pressure, the MXene could be reoriented along the surfaces of these MSSPs, then forming a kind of hollow egg‐box structure (**Figure** **1**). With the help of the hollow egg‐box structure, electromagnetic waves may occur with multiple reflections and scattering, thereby improving the composite's electromagnetic wave absorption capabilities and effectively enhancing the EMI shielding effectiveness (EMI SE) of the prepared MXene&MSPP composite (AMMF) film. Considering that MSSPs were added instead of solid particles, the specific electromagnetic shielding effectiveness per unit thickness (SSE/t) of the prepared AMMF film could further increase, which could also maintain the lightweight property of MXene.

**Figure 2:**
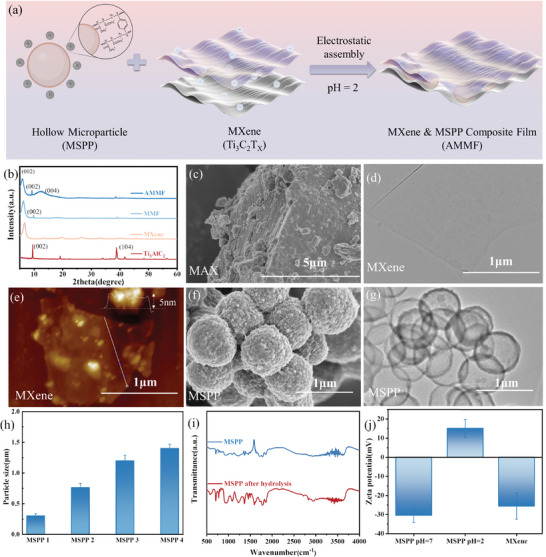
a) Schematic diagrams of self‐assembly behavior between MXene and MSSPs. b) XRD patterns of Ti_3_AlC_2_, MXene, MMF, and AMMF. c) The scanning electron microscope (SEM) image of MAX. d) TEM image of MXene. e) AFM image of MXene. f) SEM image of MSPP 3. g) TEM image of MSPP 3. h) Particle size of the prepared MSPP. i) FTIR curve of MSPP before and after hydrolysis. j) Zeta potential of MSPP dispersion (pH 2 and 7) and MXene dispersion (pH 2).

In the preparation process of MXene, lithium fluoride (LiF) and hydrochloric acid were used to selectively etch the aluminum layers from the MAX phase titanium, followed by ultrasonic dispersion to obtain monolayer MXene (Figure 1). The XRD results indicate that, compared to MAX (Ti_3_AlC_2_), the characteristic peak of the (104) plane at 38.8° disappears in MXene due to the removal of the Al layers, and the (002) diffraction peak shifts from 9.5° before etching to 6.7°, suggesting an increased interlayer spacing, and the successful transformation of MAX into MXene nanosheets (**Figure** **2**b). Additionally, as shown in Figure 2c–e, the structures of MAX and MXene reveal that after etching, MXene transitions from a multilayer stacked structure of MAX to a monolayer structure with a thickness of ≈5 nm, confirming the successful preparation of MXene nanosheets.

The preparation of MSPP is a crucial step in constructing the hollow egg‐box structure. As depicted in Figure 1, a crosslinking shell forms on the surface of the solid particles, while the uncrosslinked internal parts were removed by acetone to form a MSSP structure. As illustrated in Figure 2f,g, the prepared MPSS 3 are hollow, with an approximate diameter of 760 nm and a shell thickness of ≈50 nm, confirming the successful fabrication of MSSP. By controlling the feed ratio, we can obtained MSSPs with diameters ranging from 0.3 to 1.4 µm (Figure 2h). Figure 2i shows that after acid hydrolysis, the MSSPs showed a significant increase in the carboxylic C═O and C─O stretching peaks at 1766 and 1370 cm^−1^, indicating the conversion of anhydride groups to carboxylic acids. In Figure 2j, the zeta potential of the particles and MXene reveal that the MSSPs carry a positive charge under acidic conditions and a negative charge under alkaline conditions, while MXene carries a negative charge under acidic conditions. Therefore, under acidic conditions, the MSPP and MXene could interact via electrostatic in dispersion, subsequently forming MXene&MSPP composite (AMMF) film under the induction of vacuum‐assisted self‐assembly, as shown in Figures 2a and 1.

**Figure 3:**
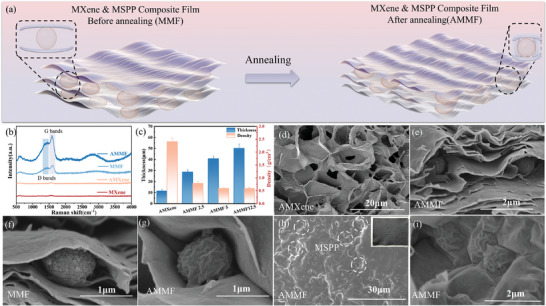
a) Schematic diagrams of MXene&MSPP composite film before and after annealing. b) Raman spectra of MXene, AMXene, MMF, and AMMF. c) Thickness and density of MXene and AMMF film. d–i) SEM image of the cross section of d) AMXene, e) AMMF film, f) MMF composite film, and g) AMMF film, h,i) SEM images of the surface of the AMMF film. A digital photograph of the prepared AMMF film is displayed in the upper right corner of panel.

In this study, AMMF film enhance their electromagnetic wave absorption capabilities through multiple reflections and scattering by the hollow egg‐box structure. Consequently, the hollow egg‐box structure of the composites prepared in this study requires further investigation. XRD and Raman spectroscopy have demonstrated successful integration of MSPP within MXene layer, and annealing under pressure may have induced bending and reorientation of the MXene on the surface of MSSPs. As shown in Figure 2b, XRD spectrum results showed that compared with MXene, the (002) diffraction peak in the MMF shifts from 6.7° to 6.2°, indicating a further increase in the interlayer distance due to the successful integration of MSPP into the MXene layers. After annealing, the 2*θ* angle of the AMMF film decreases from 6.2° to 5.8°, indicating that annealing further expands the interlayer spacing. Additionally, a new diffraction peak of AMMF film appearing at 12.8° suggests possible reorientation and bending of the MXene. Raman spectroscopy further supports these results. As shown in **Figure** **3**b, the peaks near 1450 and 1600 cm^−1^ correspond to the *D* and *G* band of graphite carbon, respectively.^[^ ^21^ ^]^ The ratio of the intensity of *D* to the *G* band reflects the proportion of disordered sp^3^ and ordered sp^2^ carbon domains, which are critical parameters for assessing the disorder and average size of sp^2^ domains.^[^ ^22^ ^]^ The absence of the D band in MXene and AMXene indicates a higher degree of order and graphitization.^[^ ^23^, ^24^, ^25^ ^]^ The I_D_/I_G_ intensity ratio of MMF is 2.06, while the I_D_/I_G_ intensity ratio increases to 2.65 in AMMF film, indicating a higher degree of disorder after annealing. The changes in the ratio may be due to that the annealing process causes the MXene layers to bend and reorient along the surface of the microparticles. Furthermore, pure MXene shows no significant change before and after annealing, further proving that the MSPP induces the reorient of MXene around the surface of MSPP. Moreover, Figure 3c shows that the thickness of the composites significantly increases after the addition of MSPP, which also confirms that the addition of MSPP increases the interlayer spacing of MXene, forming a hollow egg‐box structure. Different from the addition of other solid spheres, although the thickness significantly increases, the density of the composite material notably decreases, which is one of the advantages of the hollow egg‐box structure, as it does not influence the native lightweight characteristics of pure MXene.^[^ ^15^ ^]^

To confirm the hollow egg‐box structure in the prepared AMMF film, scanning electron microscopy (SEM) was further utilized. As shown in Figure 3d, the cross‐section of pure MXene after annealing displays a layered stacked structure, while the small‐sized monolayer MXenes stacks to form a 3D cellular structure. The internal multilevel microstructure of the prepared AMMF film in this study, as depicted in Figure 3e, is a kind of hollow egg‐box structure formed by MSSPs and MXene. Figure 3f,g presents SEM images of the cross‐sections of the AMMF films before and after annealing, respectively. These images demonstrate the successful embedding of MSSPs between MXene layers. The inherent flexibility of MXene enables it to conform to the shape of MSSPs, causing the MXene to bend along their surfaces. Furthermore, after annealing under pressure, MXene reorients and closely conforms to the contours of the MSSP surfaces. After annealing, some MXene even appeared on the surface of microparticles (Figure 3g–i) further confirm that after annealing under pressure, MXene was tightly bonded to the MSSPs.

**Figure 4:**
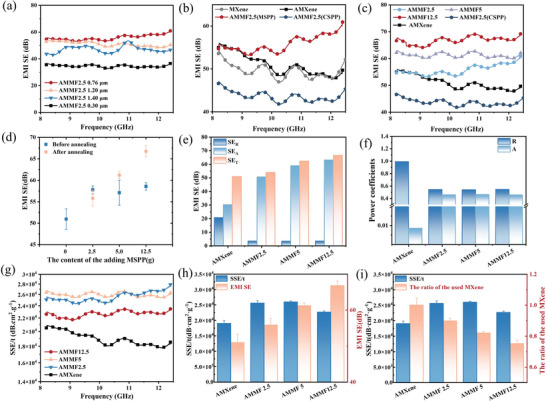
a) EMI SE as a function of frequency across different AMMF films with varying particle sizes of MSPP. b) EMI SE curve of AMMF film prepared with MSPP, MXene film by solid microparticle (CSPP) and pure MXene film before and after annealing. c) EMI SE curve of AMMF film with different content of MSPP before and after annealing. d) Effect of particle loading on the EMI SE performance of AMMF films. e,f) SE_T_, SE_A_, SE_R_, and power coefficient (A, R) of pure MXene film and AMMF film. g) The SSE/t curve of pure AMXene film and AMMF film. h) The average EMI SE and SSE/t of AMMF film. i) The average SSE/t of AMXene and AMMF film and required MXene addition for equivalent electromagnetic shielding performance.

The EMI shielding capabilities of composites with the hollow egg‐box structure were then discussed. The size of the added MSSPs significantly affected the shielding performance of the composites. As shown in **Figure** **4**a, hollow egg‐box structure with smaller microparticle in certain range could help enhance the EMI shielding ability. When using MSSPs with a diameter of 760 nm, the prepared AMMF films achieved the highest EMI shielding effectiveness, reaching 60 dB at 12.4 GHz. Consequently, microparticles with the diameter of 760 nm were utilized for further optimization of the AMMF's shielding performance in subsequent studies. To highlight the advantages of the hollow egg‐box structure over traditional ones which just add solid particles, an equal number of solid microparticles were added to fabricate conventional egg‐box structures. As illustrated in Figure 4b, the AMMF2.5 (MSPP) with the hollow egg‐box structure have a better EMI shielding ability than the traditional egg‐box structure of AMMF2.5 (CSPP). Figure 4b also shows that the traditional egg‐box structure AMMF2.5 (CSPP) exhibits lower EMI SE compared to pure MXene. In contrast, the hollow egg‐box structured AMMF2.5 (MSPP) could provide higher EMI SE than pure MXene both before and after annealing. Moreover, Figure S1 (Supporting Information) showed that annealing under pressure could increase the EMI shielding performance of the AMMF with hollow egg‐box structure, while annealing under pressure has a small effect on pure MXene film. Additionally, Figure 4c,d shows that increasing the amount of added MSSPs also enhanced the EMI shielding performance of the AMMF film. Notably, AMMF12.5 achieved the highest EMI SE of 69.19 dB at 12.4 GHz.

To further investigate the mechanisms of EMI shielding, this study analyzed the power coefficients of reflectance (R), absorbance (A), and transmittance (T), along with the overall electromagnetic shielding effectiveness (SE_T_), electromagnetic wave reflection (SE_R_), and absorption (SE_A_). Figure 4e indicates that compared with pure MXene film, the SE_R_ of the AMMF was significantly decreased, while the SE_A_ notably increased. Moreover, as depicted in Figure 4f, the R value of the AMMF composite material decreased from 0.98 to ≈0.54, while the A value increased from 0.02 to ≈0.45. Those results demonstrate that the prepared hollow egg‐box structure substantially enhances the absorption of electromagnetic waves of the AMMF, facilitating a significant reduction in immediate electromagnetic wave reflection of pure MXene.

In aerospace and microelectronics, lightweight electromagnetic shielding materials are in urgent demand. To balance both EMI shielding performance and lightweight requirements, the unit thickness electromagnetic shielding effectiveness (SSE/t) of materials has gained widespread attention due to its dependence on EMI SE, material density, and thickness. This study aims to utilize the hollow egg‐box structures which not only enhance electromagnetic wave absorption but also maintains the lightweight nature of MXene. For AMMF film, despite the thickness increased with the added MSPP, the density also significantly decreased, effectively counteracting the potential adverse effects of increased thickness on SSE/t. As illustrated in Figure 4g–i, the SSE/t of AMMF film is even better than that of pure MXene, with the AMMF2.5 film reaching a maximum SSE/t value of 27 888 dB cm^2^ g^−1^. Furthermore, as shown in Figure 4i, compared to pure MXene, the AMMF composite requires less MXene to achieve the same level of electromagnetic shielding, thereby further reducing costs, which also is one of the advantages of hollow egg‐box structure. In conclusion, for the EMI shielding property, the introduction of hollow egg‐box structures not only addresses the potential secondary electromagnetic radiation pollution caused by pure MXene, but also significantly enhances EMI SE and SSE/t performance while maintaining lightweight nature of MXene, achieving a comprehensive improvement in electromagnetic shielding performance than that of pure MXene.

**Figure 5:**
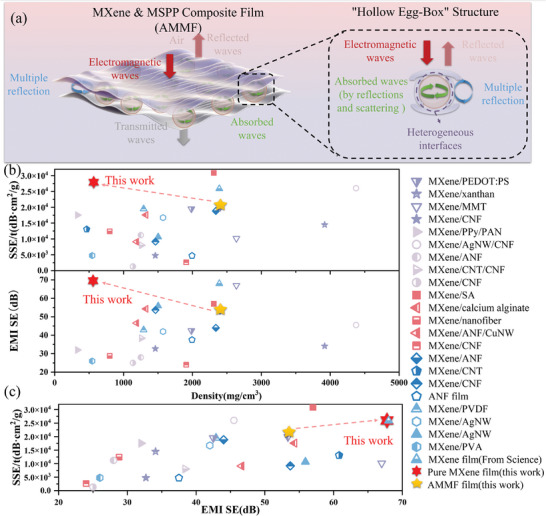
a) Schematic diagrams of the electromagnetic waves shielding mechanism in AMMF film. b) Density‐SSE/t and EMI SE curves of this work and reported MXene‐based films. c) EMI SE‐SSE/t curves of this work and reported MXene‐based film. Detailed information about the references is provided in Table S2 (Supporting Information).

In this study, we report a hollow egg‐box structure achieved by vacuum‐assisted self‐assembly and low‐temperature annealing under pressure which could achieve a comprehensive improvement than that of pure MXene. As shown in **Figure** **5**a, we attribute the effective enhancement of electromagnetic shielding performance by this structure to three main factors. Initially, when electromagnetic waves (EMW) strike the film, the impedance mismatch caused by charge carrier movement between the air and the AMMF film leads to plasma resonance, resulting in the reflection of EMWs at the film surface and blocking the majority of EMWs.^[^ ^26^ ^]^ Second, the multilevel structure of the hollow egg‐box significantly extends the transmission path for incident EMWs, causing multiple reflections and scatterings, which enhance internal scattering losses.^[^ ^27^ ^]^ Additionally, the AMMF films contain many heterogeneous interfaces (MXene‐MSPP interfaces) where charge accumulation occurs, and the functional groups present on the MXene surface undergo dipole polarization, leading to interface polarization losses.^[^ ^28^ ^]^ Ultimately, the functional groups or defects on MXene and MSPP act as dipole polarization centers. When the charge redistribution cannot match the alternating external frequency, it creates uneven charge distribution, causing polarization relaxation and enhancing polarization losses.^[^ ^29^ ^]^ Nowadays, EMI shielding materials are broadly categorized into three types: films, porous foams/aerogels. Although MXene‐based composite porous foams and aerogels can enhance MXene's electromagnetic wave absorption performance and achieve exceptional lightweighting, their typically higher thickness can also somewhat impact on the material's SSE/t, therefore limiting their use in applications. As shown in Table S1 (Supporting Information), although AMMF films in our work have a higher density than those foams/aerogels, the EMI SE and SSE/t of AMMF film are higher than that of most foams/aerogels. For most reported film materials, they can achieve thinner thickness, however, their electromagnetic shielding performance and SSE/t are commonly lower than that of pure MXene film, and typically require increased thickness to achieve satisfactory electromagnetic shielding performance. However, as depicted in Figure 5b,c, the hollow egg‐box structure could help AMMF film balance thickness and density, achieving a high SSE/t, achieving a comprehensive improvement than that of pure MXene while also ranking the top among the reported MXene composite films. Considering that the prepared AMMF films not only maintain the lightweight property but also have better performance in electromagnetic shielding performance than most of other reported MXene materials including film, foams and aerogels, which enables the fabricated AMMF film to have a broader range of applications, particularly in the field of aerospace microelectronics.

As for MXene composite film, a kind of promising candidate for advanced EMI devices in future lightweight materials, the electrical conductivity and thermal diffusivity are as vital important. The electrical conductivity of the composite films at room temperature was measured using the standard dual‐electrometer four‐point probe technique (RTS‐9, China). Results shown in Figure S2a (Supporting Information) indicate that even with the addition of MSSPs, the electrical conductivity of the MXene films remains relatively unchanged. This suggests that the incorporation of MSSPs does not significantly hinder the conductivity of the MXene layers. Additionally, the thermal conductivity of the AMMF films were tested. As shown in Figure S2b (Supporting Information), the in‐plane thermal conductivity of AMMF5 was calculated to be 46 W (m·K)^−1^, while the through‐plane thermal conductivity was 0.62 W (m·K)^−1^. The difference of thermal conductivity between the two directions indicates that the AMMF5 material is optimized for thermal management. It effectively channels heat in the desired direction for dissipation while restricting heat flow in directions where it is not needed. To visually assess the thermal conductivity of the MXene and AMMF films, both films were placed simultaneously on a heating stage (100 °C), and infrared thermal imaging was used to monitor the surface temperature changes. As shown in Figure S3 (Supporting Information), the MXene film reached 100 °C in ≈80 s, while the AMMF film took ≈100 s to reach the same temperature (Figure S3, Supporting Information). This indicates that adding fillers slightly reduced the material's thermal conductivity, but it still maintains good thermal performance. Thermal stability analysis of MSPP, MXene, and MXene/MSPP composite films showed that the thermal stability was significantly enhanced with the incorporation of MSPP microparticles into the MXene layers, with no significant loss before 230 °C (Figure S2c, Supporting Information). After annealing process, the water contact angle of the AMMF films reduced from 130° to 88°, indicating that the annealing process decreased the hydrophilicity of the composite films. As shown in Figure S2d (Supporting Information), AMMF films exhibit improved mechanical performance in terms of Young's modulus and strength. However, with the increasing amount of microparticles, both the strength and tensile modulus of the films decrease. Regarding the strain at break, only AMMF 12 have a higher strain at break compared to pure MXene film. Commonly, pure MXene film has poor water resistance,^[^ ^30^ ^]^ while the AMMF e film have an improved water resistance (Figure S4, Supporting Information), indicating that the hollow egg‐box structure also could contribute to the improved water resistance of MXene.

## 3 Conclusion

This work reveals a kind of hollow egg‐box structure used in MXene composite film by vacuum‐assisted self‐assembly and low‐temperature annealing under pressure. This structure enhances the electromagnetic wave absorption capabilities of the composite by increasing multiple reflections and scattering within the egg‐box structure. This AMMF film significantly reduces the reflectivity (R) from 0.98 to ≈0.54 and increases the absorbance (A) from 0.02 to ≈0.45, effectively decreasing the electromagnetic reflectivity of pure MXene. Concurrently, there is a significant enhancement in EMI shielding performance, with the prepared composite material AMMF 12 achieving a peak EMI SE of 69.19 dB at 12.4 GHz, which is 1.3 times that of pure MXene. Most importantly, the specific electromagnetic shielding effectiveness per unit thickness (SSE/t) of the AMMF material is not affected by the mass and thickness increase caused by the addition of fillers, and the SSE/t has improved compared to pure MXene. The SSE/t of the composite material AMMF2.5 reaches 27 888 dB cm^2^ g^−1^ at 12.4 GHz, which is 1.4 times that of pure MXene. Therefore, the hollow egg‐box structure could help AMMF film achieve a comprehensive enhancement of electromagnetic shielding performance beyond pure MXene, and the prepared AMMF films also exhibit outstanding performance among most reported MXene film, foams and aerogels, achieving a balance between material lightweight and electromagnetic shielding performance. Moreover, the AMMF films exhibit improved water resistance due to the hollow egg‐box structure. All the results showed that AMMF film could be a promising candidate for advanced EMI devices in future lightweight materials.

## 4 Experimental Section

### Materials

Ti_3_AlC_2_ powder (MAX, 200 mesh) was acquired from FoShan XinXi Technology Co., Ltd. Azodiisobutyronitrile (AIBN) and divinylbenzene were sourced from Macklin (China). HCl (12 m), isopentyl acetate, and acetone were provided by Sinopharm Chemical Reagent Co., Ltd. Lithium fluoride (LiF), heptane, maleic anhydride, and styrene were obtained from Shanghai Titan Scientific Co., Ltd.

### Preparation of Ti_3_C_2_T_x_ MXene

The synthesis of Ti_3_C_2_T_x_ (MXene) nanosheets employed a widely used gentle chemical etching process.^[^ ^31^ ^]^ Initially, lithium fluoride (LiF, 3.2 g) were dissolved in hydrochloric acid (HCl, 40 mL, 9 mol L^−1^) and stirred at room temperature for 30 min. Subsequently, MAX (Ti_3_AlC_2_) powder (300 mesh, 2.0 g) was gradually added to the LiF/HCl solution. The mixture was continuously stirred at 40 °C for 48 h, with a stirring speed set at 300 rotations per minute. Upon completion of the reaction, Ti_3_C_2_T_x_ (MXene) was obtained. For the purification of MXene, it was collected and washed with HCl (2 mol L^−1^). The washing process included centrifugation at 5000 rpm for 1 min, repeated three times to eliminate unreacted LiF. Subsequently, deionized water was used for additional washing at 5000 rpm for 1 min, repeating the process until the pH reached ≈6. To obtain monolayer MXene nanosheets, layered MXene was dispersed in deionized water and subjected to ultrasonication (power 630 W, duration 5 min), while being cooled with an ice‐water bath. The resultant mixture was then centrifuged at 3500 rpm for 15 min. The supernatant obtained post‐centrifugation constituted the monolayer MXene.

### Synthesis of the Hollow Microparticles

Hollow microparticles (MSPP) were prepared by etching core–shell crosslinked microparticles.^[^ ^32^ ^]^ Different particle sizes of MSPP can be obtained by adding different contents of solvent, monomers, and initiators, as detailed in **Table** **1**. For example, in the preparation of MSPP 3, the following steps were followed: In a 100 mL three‐necked flask, maleic anhydride (MAH, N, 2.45 g), azobisisobutyronitrile (AIB 37.5 mL), and isoamyl acetate (25 mL) were added and dispersed via ultrasonication. Subsequently, styrene (St, 1.3 g) was added and sonicated until all solids were completely dissolved. Nitrogen (N_2_) was introduced into the system and maintained for 30 min, followed by a reaction at 75 °C for 1.5 h. Then, n‐heptane (12.5 mL), divinylbenzene (DVB, 1.1 g), and azobisisobutyronitrile (AIBN, 0.005 g) were added, and the mixture was stirred at 500 rotations per minute at 75 °C for 3 h to obtain a dispersion of solid microparticles (CSPP). After adding acetone (30 mL), the core–shell particle suspension was stirred for an additional hour. To obtain MSSP, the core template was then removed through three rounds of centrifugation using acetone.

**Table 1:** Raw material feeding the amount of hollow microparticles with different particle sizes.

Sample	MAH[g]	St [g]	DVB [g]	AIBN [mg]	Isoamyl acetate [mL]	n‐heptane [mL]
MSPP 1	0.613	0.325	0.265	0.3	25	12.5
MSPP 2	2.452	1.3	1.06	0.375	25	12.5
MSPP 3	4.904	2.6	2.12	0.4	45	25
MSPP 4	7.356	3.9	3.18	0.4	45	25

### Preparation of AMMF Films

MSSP (2.5, 5, and 12.5 mg) were dispersed in deionized water (25 mL). Then, hydrochloric acid (12 mol L^−1^, 40 µL) was added to the dispersion, followed by ultrasonication (630 W for 30 min) to facilitate the acid hydrolysis of the MSPP. Subsequently, monolayer MXene dispersion (1 mg mL^−1^, 25 mL) was added, and ultrasonication was extended for another 30 min. Subsequently, the obtained mixture was filtered using a vacuum‐assisted self‐assembly device to prepare composite films named as MMF2.5, MMF5, and MMF12.5. For comparative purposes, a control sample of a pure MXene film was prepared using the same filtration process. All films were then annealed at 100 °C under the pressure of 2 MPa for 1 h, resulting in annealed films labeled as AMMF2.5, AMMF5, AMMF12.5, and AMXene, respectively.

### Scanning Electron Microscope

The structure of the MSPP and AMMF composite films was observed using SEM. The sample preparation method is as follows: AMMF composite films both before and after annealing were predried in an oven, and then, the composite films were cryofractured in liquid nitrogen, and the fractured surfaces were used as the test surfaces. The MSPP powder after freezing drying was just put on conductive adhesive. All samples were sputter‐coated with gold prior to observation.

### Transmission Electron Microscope

A dispersion of MSSPs and MXene at a concentration of 0.005 wt% was prepared and then deposited onto a transmission electron microscope (TEM) copper grid. The microstructural characterization of the MXene sheets and MSSP was conducted using a TEM (JEM‐2100PLUS).

### X‐Ray Diffraction

X‐ray diffraction (XRD) patterns of MAX, MXene, and annealed MXene/MSPP composite films were examined using an X‐ray diffractometer. The testing parameters were set as follows: the samples were scanned at a rate of 10° min^−1^ under X‐rays with a wavelength of *λ* = 0.1548 nm, covering a diffraction angle range from 5° to 60°.

### Fourier Transform Infrared Spectra

Fourier transform infrared spectra (FTIR) was conducted to analyze the MSPP before and after hydrolysis. For the preparation, the sample (1 mg) was combined with potassium bromide (250 mg) in an agate mortar. The mixture was ground clockwise for 30 s. After grinding, the mixture (130–150 mg) was uniformly placed into a mold for pressing. The pressing was performed at a pressure of 20 MPa for a duration of 2 min. The pellet was then removed and subjected to testing. The test parameters were set as follows: scanning range of 4000–650 cm^−1^, resolution of 4 cm^−1^, and the number of scans was 64.

### Zeta Potential Test

The surface charges of the prepared MSSPs and MXene were tested. MSSPs were dispersed in aqueous solutions at pH 7 and in hydrochloric acid solutions at pH 2. MXene was dispersed in aqueous solutions at pH 2. Dispersions of varying concentrations were prepared, and each sample underwent five testing cycles, the average values from these cycles.

### Raman Spectra

The Raman spectra of MXene and AMMF composite films, both before and after annealing, were examined using a micro‐confocal Raman spectrometer. The test parameters were set as follows: an excitation wavelength of 785 nm and a scanning range of 100–4000 cm^−1^.

### Electromagnetic Shielding Effectiveness Test

The EMI SE, shielding effectiveness reflection (SE_R_), and shielding effectiveness absorption (SE_A_) of film samples with a diameter of 4 cm were tested in the X‐band range (8.2–12.4 GHz) using the Dingrong DR‐S Material Shielding and Electromagnetic Parameters Test System along with a waveguide fixture.

### Statistical Analysis

Before the test, the sample quality was checked to obtain reliable raw data. Quantitative data were obtained through triplicate measurements and are presented as the mean ± standard deviation. All the graphs and charts were generated using Origin 2021 software.

## Conflict of Interest

The authors declare no conflict of interest.

## Supporting information



Supporting Information

## Data Availability

The data that support the findings of this study are available from the corresponding author upon reasonable request.
