# Supplementary material for: Lightweight MXene Composite Films with Hollow Egg‐Box Structures: Enhanced Electromagnetic Shielding Performance Beyond Pure MXene
Source: Adv Sci (Weinh). 2025 Jan 15;12(10):2411932. doi: 10.1002/advs.202411932 (PMC11904940; doi:10.1002/advs.202411932)
Supplement: Supplementary file 1 — Supporting Information [file ADVS-12-2411932-s001.docx]

Supporting Information

**Lightweight MXene Composite Films with Hollow Egg-box Structures: Enhanced Electromagnetic Shielding Performance Beyond Pure MXene**

*Yijie Wang, Chenxiang Zhao, Yunze Tian, Yue Sun, Mengfei Zhang, Kangjing Wang, Bihua Xia, Yang Wang, Ting Li, Xuhui Zhang, Jing Huang, Shibo Wang, Weifu Dong ^*^, Jinliang Qiao ^*^*

Y. Wang, C. Zhao, Y. Tian Y. Sun, M. Zhang, K. Wang, B. Xia, Y. Wang, T. Li, X. Zhang, J. Huang, S. Wang, W. Dong^,^

The Key Laboratory of Synthetic and Biological Colloids, School of Chemical and Material Engineering, Jiangnan University, 214122, Jiangsu, China

J. Qiao

SINOPEC, Beijing Research Institute of Chemical Industry, Beijing, 100013, China

E-mail: wfdong@jiangnan.edu.cn, [qiaojl.bjhy@sinopec.com](mailto:qiaojl.bjhy@sinopec.com)

Keywords: MXene, composite films, hollow egg-box structures, electromagnetic shielding performance.

*Thermal conductivity.* Thermal conductivity was measured by a laser flash apparatus (Netzsch LFA 467) and the thermal conductivity was calculated through the classical laser flash analytical method.

*Mechanical property.* The tensile test was performed with Tinius Olsen H25KS using 250 N load cell. The film was cut into 7-mm-width slips and the thickness of the film was measured for each sample using a digital caliper. The measurements were performed at a loading speed of 10 mm/min.

*TGA Thermal Analysis.* The thermal stability of the protein films was determined using thermogravimetric analysis (TGA) in a Mettler Toledo thermobalance (TGA/SDTA 851e, Switzerland) at a scanning rate of 20 ^°^C/min in a nitrogen atmosphere (50 mL min−1). The sample was pre-placed in the oven (80℃) for 12 hours. The samples of ∼5 mg in weight were placed in aluminum oxide crucibles and heated from 50 to 700 ^°^C.

*Conductivity Testing.* The conductivity of MXene and composite films was tested using an RTS-9 dual electrical measurement four-probe tester.

*Laser thermal conductivity meter test.* Samples were cut to the size of a one-corner coin(r = 1cm) and coated with graphite. The in-plane and through-plane thermal diffusivity of the film samples were then measured using a laser flash apparatus.

*Mechanical property test.* Using an automatic cutting machine, samples were cut into dumbbell-shaped specimens with dimensions of 40 mm × 4 mm × 0.5 mm. The testing was conducted at a temperature of 25 ± 2 ℃. Each specimen was stretched at a constant rate of 5 mm/min until fracture, during which the tensile strength, tensile modulus, and elongation at break were recorded. Each type of sample was measured five times to ensure repeatability.

*Water contact angle test.* The water contact angle of the film was measured by optical contact angle measuring instrument (OCA 40, Germany).

**Figure. S1** The EMI SE of MXene, MMF12.5, MMF5, MMF2.5, AMMF12.5, AMMF5 AMMF2.5 and AMMF2.5(CSPP).


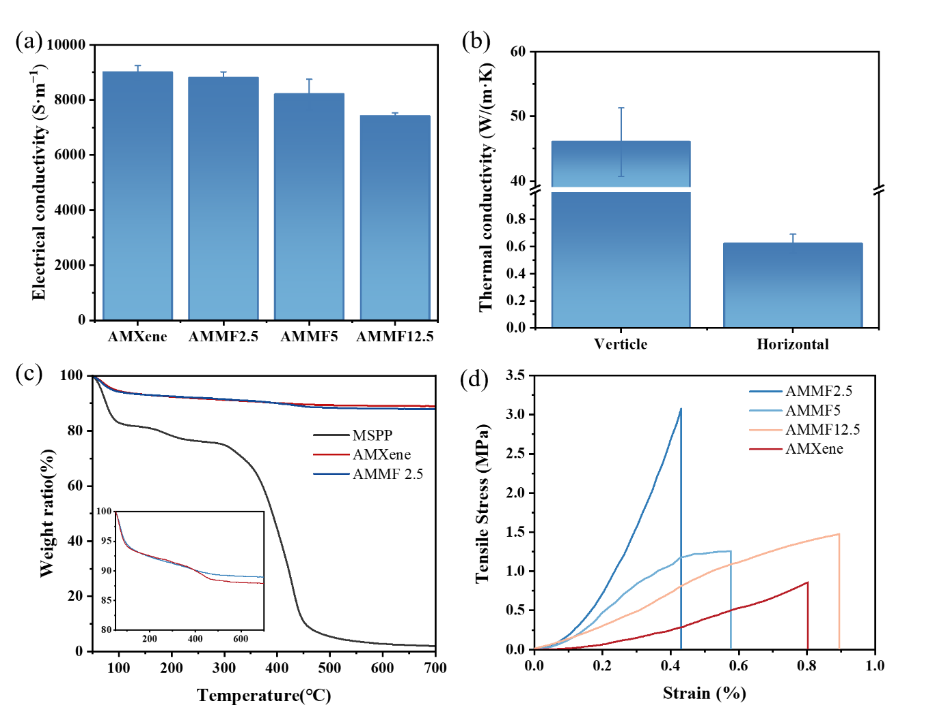


**Figure. S2** (a)The electrical conductivity of AMXene, AMMF 2.5, AMMF 5 and AMMF 12.5. (b) The thermal conductivity of AMMF composite at different directions. (c) The TGA curve of MSPP, AMXene and AMMF 2.5. (d)The tensile test of AMXene, AMMF 2.5, AMMF 5 and AMMF 12.5.


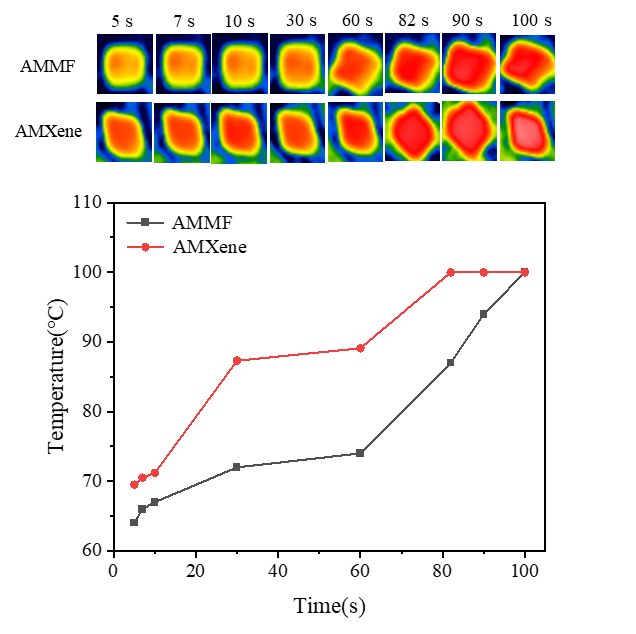


**Figure. S3** Infrared thermal images and curves of MXene heated simultaneously to 100 °C on a heating stage.


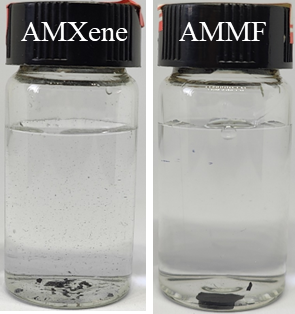


**Figure. S4** A digital photograph of the prepared AMXene and AMMF films after being immersed in 20 mL of water for 6 days.

**Table S1** EMI SE, density, thickness and SSE/t of MXene-based aerogel/foam and AMMF composite film in this work

| **Materials** | **EMI SE (dB)** | **Thickness (mm)** | **Density (mg/cm3 )** | **SSE/t (dB·cm2 /g)** |
| --- | --- | --- | --- | --- |
| MXene/cotton aerogel | 48.1 | 60 | 1.865 | 4299 |
| MXene/graphene aerogel | 75 | 29.9 | 1 | 25078 |
| MXene/WPU/Ferrite aerogel | 64.7 | 38.2 | 1.08 | 15620 |
| MXene/CNTs/Aramid aerogel | 69 | 42.8 | 2 | 8060 |
| MXene/calcium aerogel | 54.3 | 1324 | 0.026 | 17586 |
| MXene/rGO aerogel | 57.6 | 14.8 | 1.8 | 21427 |
| MXene/ANF aerogel | 56.8 | 82 | 1.9 | 3645 |
| MXene/CNF aerogel | 55.3 | 12 | 8 | 5729 |
| MXene/chitosan aerogel | 61.4 | 11.9 | 3 | 17183 |
| MXene/graphene foam | 50.7 | 8.1 | 3 | 20723 |
| MXene/carbon foam | 25 | 5 | 1.8 | 27300 |
| MXene/PU foam | 72.2 | 288.8 | 2 | 1250 |
| Ammf 2.5 film (this work) | 60.9 | 0.028 | 780 | 27888 |
| Ammf 5 film (this work) | 62.0 | 0.04 | 590 | 26267 |
| Ammf 12.5 film (this work) | 69.2 | 0.5 | 590 | 23079 |

**Table S2** SE, density, thickness and SSE/t of MXene-based reports(2D MXene-based films) and MXene pure or composite film in this work

| **Materials** | **EMI SE (dB)** | **Thickness (mm)** | **Density (mg/cm3 )** | **SSE/t (dB·cm2 /g)** |
| --- | --- | --- | --- | --- |
| MXene film from science^1^ | 68 | 0.011 | 2390 | 25863 |
| MXene/SA film^1^ | 57 | 0.008 | 2311 | 30830 |
| MXene/AgNW/CNF film^2^ | 45.5 | 0.004 | 4375 | 26014 |
| MXene/PVDF film^3^ | 42.9 | 0.017 | 1292 | 19504 |
| MXene/PEDOT:PSS film^4^ | 42.5 | 0.011 | 1985 | 19497 |
| MXene/ANF film^5^ | 44 | 0.01 | 2340 | 18847 |
| MXene/calcium alginate aerogel^6^ | 54.3 | 0.026 | 1324 | 17586 |
| MXene/PPy/PAN film^7^ | 32 | 0.055 | 331 | 17534 |
| MXene/AgNW film^8^ | 42 | 0.016 | 1573 | 16724 |
| MXene/xanthan film^9^ | 34.1 | 0.006 | 3919 | 14490 |
| MXene/CNT film^10^ | 60.8 | 0.1 | 465 | 13074 |
| MXene/nanofiber film^11^ | 28.8 | 0.029 | 800 | 12422 |
| MXene/ANF film^12^ | 28 | 0.02 | 1250 | 11200 |
| MXene/AgNW film^13^ | 55.9 | 0.035 | 1503 | 10647 |
| MXene/MMT film^14^ | 67 | 0.025 | 2638 | 10156 |
| MXene/CNF film^15^ | 53.7 | 0.04 | 1463 | 9177 |
| MXene/ANF/CuNW film^16^ | 46.6 | 0.043 | 1188 | 9120 |
| MXene/CNT/CNF film^17^ | 38.4 | 0.038 | 1260 | 8020 |
| MXene/PVA film^18^ | 26 | 0.1 | 545 | 4770 |
| MXene/CNF film^19^ | 32.7 | 0.047 | 1461 | 4761 |
| MXene/ANF film^20^ | 37.5 | 0.04 | 1994 | 4718 |
| MXene/CNF film^21^ | 24 | 0.047 | 1910 | 2647 |
| MXene/CNF film^21^ | 25 | 0.0167 | 1136 | 1326 |
| pure MXene film(this work) | 53.4 | 0.011 | 2400 | 19893 |
| Ammf 2.5 film (this work) | 57.6 | 0.028 | 780 | 27888 |
| Ammf 5 film (this work) | 62.0 | 0.04 | 590 | 26267 |
| Ammf 12.5 film (this work) | 69.2 | 0.5 | 590 | 23079 |

Reference

(1) F. Shahzad, M. Alhabeb, C. B. Hatter, B. Anasori, S. Man Hong, C. M. Koo, Y. Gogotsi, Science 2016, 353, 1137

(2) B. Wang, W. Zhang, J. Sun, C. Lai, S. Ge, H. Guo, Y. Liu, D. Zhang, Journal of Materials Chemistry A 2023.

(3) Y. Li, B. Zhou, Y. Shen, C. He, B. Wang, C. Liu, Y. Feng, C. Shen, Composites Part B: Engineering 2021, 217.

(4) R. Liu, M. Miao, Y. Li, J. Zhang, S. Cao, X. Feng, ACS Appl Mater Interfaces 2018, 10, 44787.

(5) C. Liu, Y. Ma, Y. Xie, J. Zou, H. Wu, S. Peng, W. Qian, D. He, X. Zhang, B. W. Li, C. W. Nan, ACS Appl Mater Interfaces 2023.

(6) Z. Zhou, J. Liu, X. Zhang, D. Tian, Z. Zhan, C. Lu, Advanced Materials Interfaces 2019, 6.

(7) F. Wu, Z. Tian, P. Hu, J. Tang, X. Xu, L. Pan, J. Liu, P. Zhang, Z. Sun, Nanoscale 2022, 14, 18133.

(8) M. Miao, R. Liu, S. Thaiboonrod, L. Shi, S. Cao, J. Zhang, J. Fang, X. Feng, Journal of Materials Chemistry C 2020, 8, 3120.

(9) Y. Sun, R. Ding, S. Y. Hong, J. Lee, Y.-K. Seo, J.-D. Nam, J. Suhr, Chemical Engineering Journal 2021, 410.

(10) R. Yang, X. Gui, L. Yao, Q. Hu, L. Yang, H. Zhang, Y. Yao, H. Mei, Z. Tang, NanoMicro Letters 2021, 13.

(11) Y. Wang, T.-T. Li, B.-C. Shiu, X. Zhang, H.-K. Peng, C.-W. Lou, J.-H. Lin, Applied Surface Science 2022, 574

(12) T. Hua, H. Guo, J. Qin, Q. Wu, L. Li, B. Qian, RSC Adv 2022, 12, 24980

(13) B. Zhou, Q. Li, P. Xu, Y. Feng, J. Ma, C. Liu, C. Shen, Nanoscale 2021, 13, 2378.

(14) L. Li, Y. Cao, X. Liu, J. Wang, Y. Yang, W. Wang, ACS Appl Mater Interfaces 2020, 12, 27350

(15) Z. Cui, C. Gao, Z. Fan, J. Wang, Z. Cheng, Z. Xie, Y. Liu, Y. Wang, Journal of Electronic Materials 2021, 50, 2101.

(16) F. Jia, J. Dong, X. Dai, Y. Liu, H. Wang, Z. Lu, Chemical Engineering Journal 2023, 452

(17) W. Cao, C. Ma, S. Tan, M. Ma, P. Wan, F. Chen, Nano-Micro Letters 2019, 11

(18) H. Xu, X. Yin, X. Li, M. Li, S. Liang, L. Zhang, L. Cheng, ACS Appl Mater Interfaces 2019, 11, 10198.

(19) Z. Zhan, Q. Song, Z. Zhou, C. Lu, Journal of Materials Chemistry C 2019, 7, 9820.

(20) J. Zhou, J. Yu, D. Bai, H. Liu, L. Li, Nanomaterials (Basel) 2021, 11

(21) W. T. Cao, F. F. Chen, Y. J. Zhu, Y. G. Zhang, Y. Y. Jiang, M. G. Ma, F. Chen, ACS Nano 2018, 12, 4583.
